# The Impact of Climate Change on Ozone-Related Mortality in Sydney

**DOI:** 10.3390/ijerph110101034

**Published:** 2014-01-13

**Authors:** William Physick, Martin Cope, Sunhee Lee

**Affiliations:** CSIRO Marine and Atmospheric Research, Station Street, Aspendale, Victoria 3195, Australia; E-Mails: martin.cope@csiro.au (M.C.); sunhee.lee@csiro.au (S.L.)

**Keywords:** ozone, mortality, climate change, modeling, Sydney, urban scale

## Abstract

Coupled global, regional and chemical transport models are now being used with relative-risk functions to determine the impact of climate change on human health. Studies have been carried out for global and regional scales, and in our paper we examine the impact of climate change on ozone-related mortality at the local scale across an urban metropolis (Sydney, Australia). Using three coupled models, with a grid spacing of 3 km for the chemical transport model (CTM), and a mortality relative risk function of 1.0006 per 1 ppb increase in daily maximum 1-hour ozone concentration, we evaluated the change in ozone concentrations and mortality between decades 1996–2005 and 2051–2060. The global model was run with the A2 emissions scenario. As there is currently uncertainty regarding a threshold concentration below which ozone does not impact on mortality, we calculated mortality estimates for the three daily maximum 1-hr ozone concentration thresholds of 0, 25 and 40 ppb. The mortality increase for 2051–2060 ranges from 2.3% for a 0 ppb threshold to 27.3% for a 40 ppb threshold, although the numerical increases differ little. Our modeling approach is able to identify the variation in ozone-related mortality changes at a suburban scale, estimating that climate change could lead to an additional 55 to 65 deaths across Sydney in the decade 2051–2060. Interestingly, the largest increases do not correspond spatially to the largest ozone increases or the densest population centres. The distribution pattern of changes does not seem to vary with threshold value, while the magnitude only varies slightly.

## 1. Introduction

The first USA National Assessment of the Potential Consequences of Climate Variability and Change was completed in 2000. In an update of the Health Sector Assessment of that Report [1], a review of literature published since the first assessment did not change the initial conclusion that the net health impact of climate change is uncertain. In particular, [1] state that “Perhaps most important, the continued lack of reliable local and regional climate change projections limits the ability of researchers to quantify the attributable burden of diseases due to climate change.” 

With regard to the human-health consequences of climate-induced changes in air quality, quantitative estimates have been obtained by a few researchers [2,3,4,5,6] through an approach using coupled meteorological and air chemistry numerical models. Simulations are done out to as far as the 22nd century, using greenhouse-gas emissions from scenarios covering a wide range of the main driving forces of future emissions, from demographic to technological and economic developments. The air pollution estimates are linked to health outcomes by a concentration–response function, which quantifies the magnitude of the proportional change in daily mortality or morbidity that would be expected in response to a given daily concentration of a specified pollutant.

In [3] daily surface ozone (O_3_) concentrations from a global atmospheric transport and chemistry model, and ozone–mortality relationships from daily time-series studies were used to explore the effects of projected future changes in global ozone concentrations on premature human mortality, under three scenarios for 2030. The model was run with a horizontal resolution of 3.758 degrees in longitude (typically 400 km in mid-latitudes) and 2.58 in latitude (about 280 km). Mortality effects, driven by the daily maximum 8-h average ozone concentration on each day, were calculated on this grid also.

In [2] the authors also used a global climate model (GISS GCM), but by coupling it with a regional meteorological model (MM5) and a chemistry model (CMAQ) were able to simulate hourly meteorology and ozone concentrations at a finer grid resolution of 36 km for the eastern United States. Using a dose-response relation involving the daily 1-hr maximum O_3_ concentration, they assessed changes in O_3_-related impacts on summer mortality over five summers of the 2050s decade across the 31-county New York metropolitan region. [4] utilized the same simulations by interpolating the gridded ozone concentration fields at the surface layer to the location of 50 cities in the eastern USA. Percentage increases, but not absolute numbers, in hospital admissions or mortality for a city were obtained by multiplying the increase in daily average ozone from the 1990s to the 2050s by a concentration-response function. The health impact estimates were calculated using concentration-response functions from multiple epidemiological studies, derived from data obtained in USA cities. 

In [5], an update of an earlier study [7], ozone-related mortality and morbidity changes between 2003 and 2020 for the United Kingdom under climate change and projected changes in emissions for the UK and Europe were examined. A photochemical trajectory model (OSRM) was used to calculate hourly-ozone concentrations on a 10 × 10 km grid covering the UK, although the meteorology used to calculate the 96 h back trajectories for each cell was interpolated from global datasets at a horizontal resolution of 0.83 latitude × 1.25 longitude obtained from the Hadley Centre Climate Model (HadCM3) simulations. This work has been expanded recently to the regional scale by running a photochemical model with a 5-km grid across the UK, although a climate change meteorological scenario for that grid was obtained by adding 5 °C to the temperature fields from a 2003 base-case simulation [8,9]. A similar study, but expanded to include European countries, was recently done by [6], with results suggesting that a marked variation in mortality and morbidity across Europe in the period 2041–2060 could result from projected effects of climate change on ozone concentrations.

While these studies provide guidance on the *regional* health impact of climate change, there is a need within large cities for finer spatial detail in the long-term planning associated with the allocation of health facilities and resources. Should this allocation be based on the current distribution of hotspots in emissions or in pollutant concentrations, or on the largest population groupings, and are such decisions dependent on the pollutant under consideration? To examine these questions, we propose a method that extends the use of global and regional meteorological and chemical transport models by including a mesoscale atmospheric transport and chemistry model (3-km grid spacing) that resolves the local/urban scale of a city. In this paper, we investigate the feasibility of such an approach by applying the modeling system to Sydney (Australia) to examine the impact of climate change on ozone-related mortality at the suburban scale for the decade 2051–2060. No such analyses have been done previously for Australia.

## 2. Experimental Section

Situated on the coast at latitude 34°S, Sydney is Australia's largest city with a 2006 population of 4.1 million people [10]. The city and suburbs are contained in a large basin (area 12,000 km^2^) bounded on the east by the Pacific Ocean and to the north, south and west by hills and mountains extending to over 1,000 m. Within the general basin, there are smaller basins and valleys which play important roles in the recirculation of pollutants. Sydney contains the usual industrial and commercial activities such as transport, port facilities, refineries and manufacturing industry, but its electricity is generated at power stations in rural areas more than 100 km from the city centre.

Photochemical smog episodes are often associated with sea breeze conditions, with morning emissions of oxides of nitrogen (NO_x_), carbon monoxide (CO) and volatile organic compounds (VOCs) being transported offshore by land breezes. This gaseous mixture reacts chemically to form ozone, which is then recirculated inland by the action of the sea breeze. Elevated concentrations of ozone (the principal component of photochemical smog) may also be associated with bushfires and controlled burns, with ozone generation from VOCs, NO_x_ and CO released by fires contributing to the photochemical smog generated within urban precursor plumes.

### 2.1. Modeling of Meteorology and Air Quality

The downscaling system set up for this project consists of nesting The Air Pollution Model- Chemical Transport Model (TAPM–CTM), an urban/regional atmospheric transport and chemistry model (3-km inner grid spacing; [11,12]), into the regional meteorological fields (60-km grid spacing) generated by the stretched grid model Cubic Conformal Atmospheric Model (CCAM; [13]), which in turn is nudged towards global-scale meteorological fields generated by the CSIRO–Mk3 Global Climate Model (CGCM; [14]). 

CGCM has been extensively used by CSIRO for the development of climate change scenarios in response to the requirements of the IPCC, typically being integrated for the period 1871–2100. The model includes a comprehensive representation of the four major components of the climate system—atmosphere, land surface, oceans and sea ice. The atmospheric component uses a T63 spectral grid (1.875°EW × 1.875°NS—approximately 150–200 km grid spacing in the horizontal) with 18 vertical levels with the lowest level being 165 m above ground level and the top of the model domain extending to 36,355 m above the ground. The atmospheric component is coupled directly to an ocean model, based on the GFDL MOM2 code. Additional details of the CGCM are given in [14].

In the current project we have used CGCM output generated using the A2-ASF emissions scenario. The A2 family of emissions scenario is summarised in [15] as follows. “The family represents a differentiated world. Compared to the A1 storyline it is characterized by lower trade flows, relatively slow capital stock turnover, and slower technological change. The A2 world “consolidates” into a series of economic regions. Self-reliance in terms of resources and less emphasis on economic, social, and cultural interactions between regions are characteristic for this future. Economic growth is uneven and the income gap between now-industrialized and developing parts of the world does not narrow, unlike in the A1 and B1 scenario families”.

The CGCM predictions are downscaled to the regional level using the CSIRO Cubic Conformal Atmospheric Model (CCAM; [13,16,17]). CCAM is formulated on a conformal-cubic grid which covers the globe, but can be stretched by utilising the Schmidt [18] transformation to provide higher resolution in an area of interest. Because of its higher resolution, CCAM is, in principle, able to generate more accurate climate predictions over Australia, with particular emphasis on the Sydney region where the grid spacing is 60 km. CCAM was integrated for the period 1961–2100 for the current study, primarily to create meteorological boundary conditions suitable for use with the smaller-scale model TAPM-CTM. These were generated at 6-hourly intervals for the periods 1996–2005 and 2051–2060.

TAPM-CTM is a mesoscale atmospheric modeling system with the ability to undertake numerical weather prediction and complex chemical transformation modeling. It consists of the nestable, three-dimensional Eulerian meteorological model from the air quality prediction system TAPM [11], and a three-dimensional Eulerian chemical transport model CTM [11]. CTM was run as an inline module to TAPM and was called within the main time-marching loop of the host model at 300s intervals.

For the current study TAPM-CTM was configured with three nested grids (60 × 70 rows and columns in the horizontal and centred on Sydney) with cell spacings of 12, 6 and 3 km. The model was configured with 25 levels in the vertical with levels centred on the following heights (m): 10, 25, 50, 100, 150, 200, 250, 300, 400, 500, 600, 750, 1,000, 1,250, 1,500, 1,750, 2,000, 2,500, 3,000, 3,500, 4,000, 5,000, 6,000, 7,000 and 8,000. Boundary conditions for the 12–km TAPM-CTM grid were generated by linear interpolation of 6-hourly CCAM wind, ambient temperature, specific humidity and sea surface temperature fields. The boundary conditions for the 6 km and 3 km grids were generated from corresponding 12 km and 6 km TAPM simulations and updated at 15 min intervals. In this way, atmospheric climate change as modelled by the CGCM and downscaled by CCAM is able to force the mesoscale flows (sea breezes, katabatic and anabatic winds) that influence air quality within the Sydney basin.

Motor vehicle emissions for the study were based on the Sydney Greater Metropolitan Region (GMR) on-road mobile source inventory [19]. Industrial, commercial and domestic emissions were taken from the Metropolitan Air Emissions Inventory [20]. Emissions from natural sources (NO_x_ from bacterial activity in soils; VOC emissions from plants) were also modelled (see [21] and references therein). This inventory set for the 2003 calendar year was used for both decades in order to isolate the impact of climate change alone on ozone levels and health. However, it should be noted that anthropogenic and natural emissions of VOCs, NO_x_ and CO were allowed to vary with ambient temperature on an hourly basis.

CCAM model results at 60 km resolution have been validated across Australia for the period 1960–1990 [22]. The downscaling captures the location of the centre of circulations and the broad spatial distribution of maximum daily temperature very well, correcting a cold bias in the CGCM simulation. Evaluation of further downscaling of the CCAM results to a 3 km spatial resolution by TAPM-CTM was done by comparing model output for the period 1998–2005 with data from four monitoring stations approximately located in a line of increasing distance from the coast. For each warm-season month, the modeling system was able to reproduce the observed large spatial gradients in peak daily temperature within the vicinity of Sydney’s coastline. As well, TAPM-CTM generally did a reasonable job at reproducing the frequency distributions of near-surface wind speed and direction.

The performance of the downscaling system with respect to ozone concentrations was explored further by examining scatter plots of the observed and modelled peak 1 and 4 h concentrations for various percentiles of the frequency distributions for 16 air quality stations sited around the Sydney region [22]. The distributions have been generated for the periods January–March and October–December inclusive for 1998–2005. The majority of the modeled concentrations lie within 30% of the observed concentrations, although the model has a tendency to over predict the lower percentile concentrations and to under predict the upper percentile concentrations. In summary, the system was found to perform well in the prediction of the historical ozone climatology, mesoscale meteorology and peak ozone concentrations.

### 2.2. Health Impact Analysis

Short-term acute effects of ozone include respiratory symptoms, pulmonary function changes, increased airway responsiveness and airway inflammation. Higher concentrations, longer exposure duration and greater activity levels during exposure are known risk factors. Ozone exposure has been reported to be associated with increased hospital admissions for respiratory causes and exacerbation of asthma, and more recently with premature mortality—see [23] for a review of ozone-related mortality studies.

In our study, we have chosen to focus on the mortality impact associated with climate-induced changes in ozone levels, as modelled for Sydney by [22], by calculating mortality numbers for each decade separately. We use a risk-assessment approach based on concentration-response functions (in the form of a relative risk) derived from epidemiological studies. For each day of a decade, the number of ozone-related deaths ∆A is calculated at each gridpoint from:

∆A/A = (RR-1)∆C
(1)

where A is the daily total all-cause mortality (excluding accidental and other external causes), RR is the relative risk per 1 ppb increase in ozone concentration, and ∆C is the amount by which the daily maximum 1-hour ozone concentration exceeds a threshold value, below which ozone is assumed to have no effect on mortality. Equation (1) is equivalent to that used in the New York study by [2]. The deaths per decade are obtained by adding the daily deaths. We restrict our calculations to the warmer months (October–March (in the next year)) and examine mortality figures for 1996–2005 and 2051–2060. Equation (1) is applied at each gridpoint of the 3 km × 3 km modeling grid and the ∆A values are summed across the grid to obtain the total ozone-related deaths for Sydney.

The Australian Bureau of Statistics mortality data used in our study is the number of deaths not due to external causes in the Sydney Statistical Division from October 2004 to March 2005. For our study, this value (10,931 deaths) is distributed across the modeling grid in proportion to the population in each grid square. The latter is obtained by interpolation from the 2001 Statistical Local Area (SLA) Census data set. There are 41 SLAs in the Sydney urbanized area, covering 3700 km^2^ and encompassing 4 million people. Note that the same population distribution is used in the analysis of the 2051–2060 ozone concentrations, in order to isolate the impact of climate change on ozone-related mortality.

### 2.3. Choice of Relative Risk Value

The concentration-response function, and associated relative risk value, used in our study is that derived by [24] from an analysis of data from the Air Pollution and Health: A European Approach study (APHEA2) in which daily ozone concentrations and daily mortality statistics were collected for 23 European cities/areas over a period of at least 3 years. Their analysis found no significant links between ozone and short-term mortality during the cold half of the year, but for the warm season, an increase in the daily maximum 1-hour ozone concentration by 10 µg/m^3^ (5 ppb) was associated with a 0.33% (95% confidence interval (CI), 0.17–0.52) increase in the total daily number of deaths. The corresponding figures for the maximum 8-hour ozone concentration were similar. Our analysis uses daily maximum 1-hour ozone concentrations for each day of 1996–2005 and 2051–2060 with a value of 1.00066 for the relative risk per 1 ppb increase in ozone concentration.

Corresponding RR values used in other ozone and climate-change mortality studies include 1.00056 by [2], taken from a pooling of seven studies that controlled for temperature effects using nonlinear functions, 1.00052 by [3], following the analysis of [25], and 1.0006 by [5] from a meta-analysis that combined the APHEA2 results with those from four additional European cities. A slightly different approach was taken by [4] who used values from seven different studies, ranging from 1.00038 to 1.00094.

An epidemiological study by [26] of Sydney air quality and health data (all months during 1989–1993) derived a percentage increase in all-cause mortality of 2.04% associated with an increase of ozone concentration from the 10th (13 ppb) to the 90th percentile (41 ppb). This converts to 1.00073 per 1 ppb.

In a study examining the long-term chronic effects of ozone on mortality, [27] analysed data over a 23-year period from 96 metropolitan statistical areas in the United States, and found no statistically-significant effect of warm-season ozone on all-cause mortality. In this study, for each year, a single value for exposure was used for all participants in a metropolitan area, calculated as the average of daily 1 h maximum concentrations over the warm season averaged over all monitors in the area. Note however that the short-term relative risk factors used in the papers referenced in this section were derived from statistical analyses using time series of daily concentrations and mortality over short-term periods of typically 3–5 years.

### 2.4. Threshold Values

There is currently uncertainty regarding the value, or even existence, of a threshold concentration below which ozone does not impact on mortality. In a study of Rotterdam, The Netherlands [28] found that relative risk estimates of mortality associated with daily changes in O_3_ were robust to exclusion of days with a 24 h average ≥ 40 μg/m^3^ (20 ppb), concluding that should a threshold exist, it may be at a low concentration. [29], using a spline model approach and 5 years of data from Seoul, estimated a threshold of around 20–30 ppb for the daily 1-hr maximum, approximately equal to 8–12 ppb for the 24 h average. For the summer season, a value of approximately 40 ppb was found for the daily 1 h maximum. Using a data set covering 98 U.S. urban communities, [30] used the average of the same day and previous day 24 h averages of ozone concentration to examine concentration-exposure relations for various models that took into account different thresholds. They found that daily changes in ambient O_3_ were significantly associated with daily changes in the number of deaths, even when they used data that included only days with the two-day average O_3_ levels <15 ppb. Their work suggested that a “safe” level is <10 ppb. Discussion of further epidemiological, and clinical, studies related to thresholds can be found in the review of the national ambient air quality standards for ozone [31], which concludes that “there is insufficient evidence to support use of potential threshold levels in quantitative risk assessments and that it is appropriate to estimate risks within the range of air quality concentrations down to estimated policy-relevant background level”. 

In our study for the six warmer months of the year, we use time series of daily maximum 1 h ozone concentrations for each decade to calculate mortality estimates for the three concentration thresholds of 0, 25 and 40 ppb. In this way, numbers are obtained for the summertime estimate (40 ppb) of [29], the lowest possible threshold value of zero and an in-between value of 25 ppb. For each day, the threshold value is subtracted from the daily 1-hr maximum, with negative results being set to zero. As [24] found that their dose response curve for total mortality effects does not deviate significantly from linearity across the range of ozone concentrations, there is no need to adjust the relative risk value for the different threshold values.

## 3. Results

**Figure 1:**
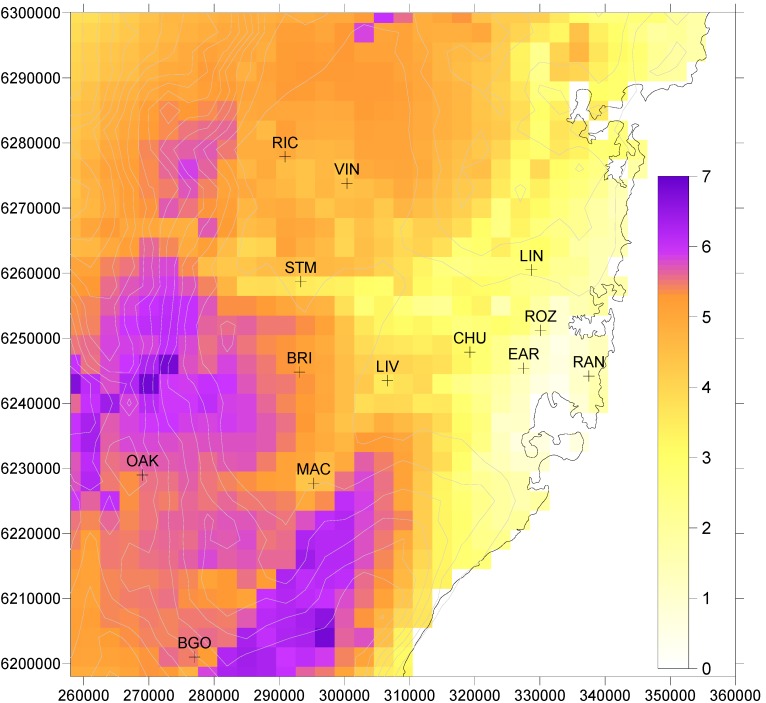
Daily 1 h maximum ozone concentration (ppb) averaged over the period 1996–2005, with a threshold value of 40 ppb subtracted (negative values are set to zero). Contours indicate elevation above sea level at 50 m intervals up to 850 m. Symbols indicate locations of air quality stations in the EPA New South Wales monitoring network. Values on axes in all Figures are distance in meters.

Figure 1 shows the distribution of the daily 1 h maximum ozone concentration, with a threshold value of 40 ppb subtracted, averaged over the period 1996–2005. The largest values are found to the southwest of Sydney and this is consistent with the regular sea-breeze transport of urban emissions to this area by the end of the day [22]. Highest ozone concentrations are found here as the urban plume has sufficient NO_x_ to continue ozone production while there is still daylight. Titration of ozone by NO_x_ sources in the metropolitan area also contributes to the lower concentrations nearer to the coastline.

**Figure 2:**
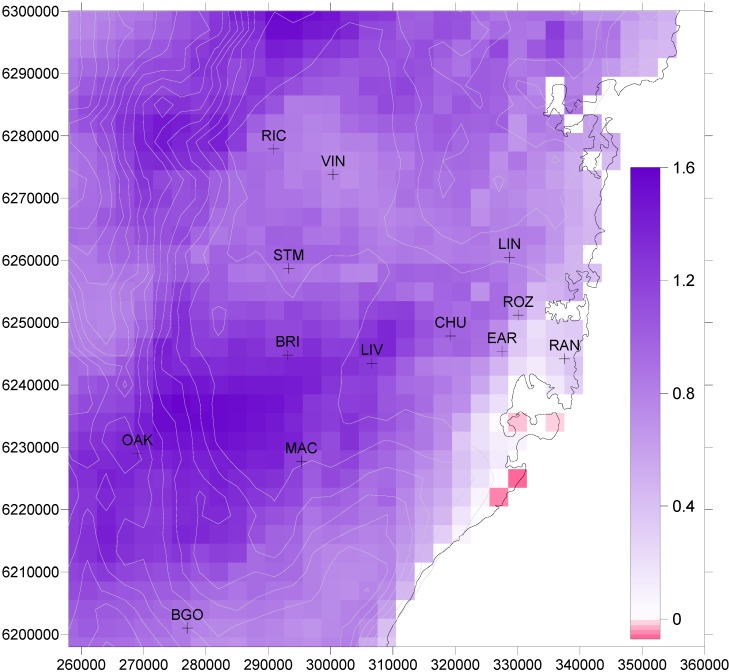
Difference between daily 1 h maximum ozone concentration (ppb) averaged over the periods 2051–2060 and 1996–2005.

The change in the values from 1996–2005 are shown in Figure 2 for 2051–2060, with positive values found everywhere, apart from in small coastal areas. Analysis by [22] suggested that the most significant single factor responsible for the increase in ozone concentration and extent for 2051–2060 is the increased frequency of hot days, and hence the conditions which are conducive for photochemical smog generation downwind of Sydney. The small reduction in motor vehicle NO_x_ and increases in VOCs from evaporative vehicle and natural sources, together with more rapid rates of photochemical transformation at higher temperatures, are the main causes of increases in peak ozone concentration in 2051–2060. The largest increases in ozone do not coincide with the highest concentrations for the 1996–2005 decade, but are found to the northwest of that region. In the later decade, ozone concentrations are predicted to be equally as high in both areas. While a small change in wind patterns on high ozone days could be responsible, comparison of frequency distributions of wind speed and wind direction for both decades at various sites by [22] indicated that there is not a significant change in the mesoscale flows generated for each decade.

The decadal ozone mortality statistics under each threshold assumption are listed in Table 1. The importance of further research to reduce the uncertainty in the threshold concentration below which there are no mortality effects is illustrated by the wide range in predicted deaths, with an order of magnitude between thresholds 0 and 40 ppb. For a zero threshold, the increase in mortality by 2051–2060 is 2.3%. The only other comparable study [2] found a 4.5% increase for the New York metropolitan region, although a 36 km grid spacing was used in that work.

**Table 1:** Ozone-related mortality statistics for each decade and threshold assumption.

	Threshold 40 ppb		Threshold 25 ppb		Threshold 0 ppb	
	1996–2005	2051–2060	1996–2005	2051–2060	1996–2005	2051–2060
Ozone mortality	201	256	795	860	2571	2631
% increase over 1996–2005 mortality		27.3		8.1		2.3

## 4. Discussion

From Table 1, the annual deaths for Sydney averaged over the period 1996–2005 are estimated to be 20, 79 and 257 for the thresholds 40, 25 and 0 ppb, respectively. This differs greatly from the Australian ozone National Environment Protection Measure (NEPM), which assumes annual mortality in all of Australia due to ozone exposure is about 5–10 [32]. Apart from some differences in methodology between our work and the NEPM document, the biggest discrepancy in mortality arises from the assumed threshold concentrations. A value of 80 ppb is used in [32] and although uncertainty in the threshold concentration still exists, it is clear from the more-recent research discussed earlier in our paper that 80 ppb is no longer a valid choice.

The numerical increase in decadal deaths for Sydney between 1996–2005 and 2051–2060 is very similar for each threshold value: 55, 65 and 60 for the 40, 25 and 0 ppb thresholds respectively. This follows from there being little difference between the decadal increase in daily 1 h maximum ozone concentration, averaged over populated gridpoints, for each threshold value: 0.89, 1.05 and 1.01 ppb respectively. It further follows that the increase in daily maximum ozone concentrations must be distributed fairly evenly across the bands 0–25 ppb, 25–40 ppb and above 40 ppb. 

Interestingly, the opposite conclusion can be made from the study of climate change-related ozone mortality across the United Kingdom (UK) for 2020 [5], in which differences from 2003 in ozone-related mortality of 177, 810 and 1658 were predicted for thresholds of 50, 35 and 0 ppb—the same order of magnitude difference was also found by [8]. The important difference between our study and that of [5] is that the latter incorporated projected emissions for 2020 across Europe and the UK, whereas our emissions were kept constant for all decades. The same dependence of mortality change on threshold value was found by [5] in a simulation in which projected 2020 emissions were used with the 2003 climate. This occurs because emission changes can cause changes in ozone concentrations, especially through titration by NO_x_, that are larger than those caused by climate change through higher temperatures and perhaps modifications to mesoscale wind systems. It also underlines the importance of modeling the projected changes for individual emission sources at the local scale across an urban area.

**Figure 3:**
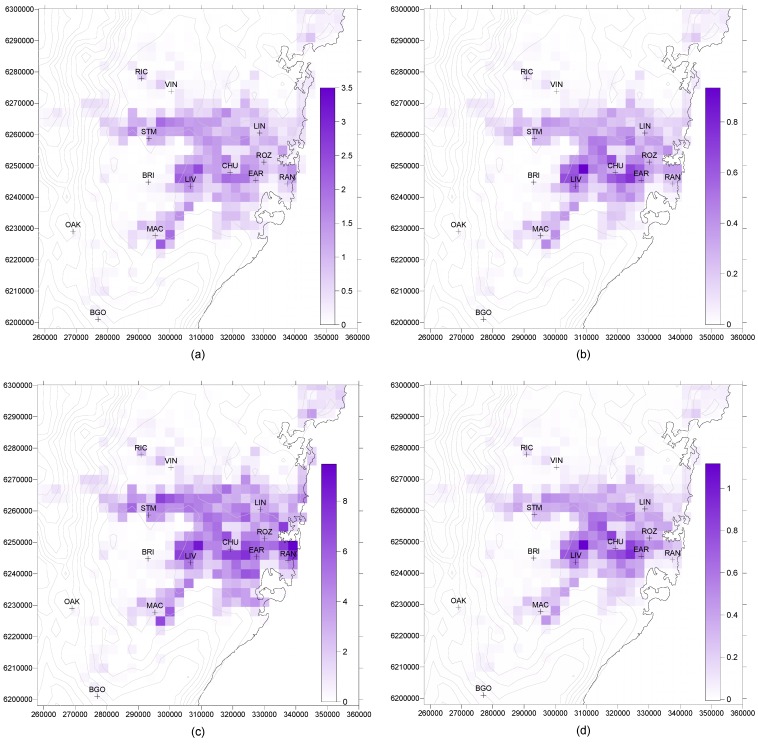
(**a**) Ozone-related mortality (deaths per 9 km^2^) for 1996–2005. A threshold ozone concentration of 40 ppb for mortality has been assumed. (**b**) Increase in mortality (deaths per 9 km^2^) for 2051–2060 over mortality in 1996–2005. A threshold ozone concentration of 40 ppb for mortality has been assumed. (**c**) Ozone-related mortality (deaths per 9 km^2^) for 1996–2005. A threshold ozone concentration of 25 ppb for mortality has been assumed. (**d**) Increase in mortality (deaths per 9 km^2^) for 2051–2060 over mortality in 1996–2005. A threshold ozone concentration of 25 ppb for mortality has been assumed.

**Figure 4:**
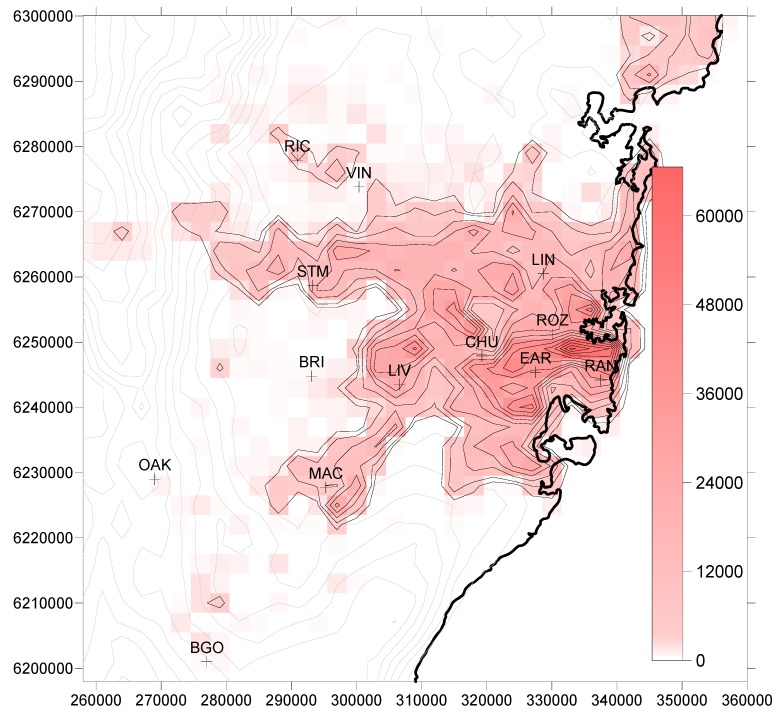
Population distribution across the Sydney metropolitan area according to 2001 census data.

The mortality pattern across Sydney for a threshold of 40 ppb, shown for 1996–2005 in Figure 3a, is not the same as the ozone distribution of Figure 1. This is because of the population distribution, shown in Figure 4, which demonstrates that areas of highest ozone concentration do not coincide with the most dense population centres. Nor does the highest mortality occur in the densest population region near the coast (where there are relatively low ozone concentrations), but is further inland towards Liverpool (LIV) where *both* population and ozone are of reasonable magnitude.

The distribution of increased mortality above the 1996–2005 decade is shown in Figure 3b for 2051–2060. While the largest increases occur in an east-west strip from Liverpool across to Earlwood (EAR), they are not coincident with the highest ozone increases between the two decades (Figure 2), which are found to the southwest of this region, where population distribution is sparse.

Mortality with a threshold of 25 ppb (Figure 3c) is of course higher than for 40 ppb, and an interesting effect is that the densely-populated eastern suburbs now differ little from Liverpool as the area of highest mortality. The pattern of mortality differences between the two decades in Figure 3d is similar to that for a threshold of 40 ppb (Figure 3b), and to that for a 0 ppb threshold (not shown).

While the results presented in Table 1 are subject to uncertainties associated with the chosen RR value (see Section 2.3), further uncertainties arise from other components of the methodology presented here. Different global emissions scenarios and global climate and chemistry models, different projections for local air emissions and populations, and alternative concentration-response functions (CRFs) will all lead to varying results. An analysis by [33] of ozone-related human health impacts estimated from combinations of various models (seven), population projections (five) and several CRFs found that estimates for the United States of America ranged from 2,500 deaths down to 600 deaths actually avoided as a result of climate change. The choice of the climate change and the air quality model reflected the greatest source of uncertainty, with the other modeling choices having lesser but still substantial effects. Their results suggest the need to use an ensemble approach, rather than a single set of modeling choices, to assess the potential risks associated with O_3_-related human health effects resulting from climate change.

## 5. Conclusions

We conclude that climate change is estimated to cause an additional 55 to 65 deaths (above current levels) in the decade 2051–2060. The effects of uncertainty in the value of a threshold concentration, below which there is no ozone-related mortality, have been highlighted by our work. While there is an order of magnitude difference in the predicted number of deaths in either decade according to whether a threshold value of 0 or 40 ppb is chosen, there is little difference for each threshold in the mortality changes between decades. The distribution pattern of mortality increases across Sydney in 2051–2060 does not appear sensitive to an assumed threshold value.

The importance of including urban-scale transport and chemistry models in our methodology has been illustrated by the Sydney case study results, which show that planning-related issues such as the distribution and magnitude of climate change-related ozone mortality cannot be determined a priori by consideration of existing population density and emission or concentration hotspots. This is without the further complications of projected urban emissions and populations, which have not been considered in our simulations (kept constant in order to isolate the impact of climate change alone), but which can be easily incorporated in our modeling system. Additionally, the same methodology can be applied to other health-impacting pollutants such as particle matter, nitrogen dioxide and toxic volatile organic compounds.
